# Study on the influence of balloon dilation mode on the intravertebral cleft of osteoporotic fracture

**DOI:** 10.1186/s12893-022-01750-5

**Published:** 2022-09-30

**Authors:** Nanning Lv, Xiaoxiao Feng, Haojun Liu, Xuejun Jia, Shanqin Han, Mingming Liu

**Affiliations:** 1 Department of Orthopedic Surgery, Bengbu Medical College Lianyungang Clinical College, The Second People’s Hospital of Lianyungang, 41 Hailian East Street, Lianyungang, 222003 Jiangsu China; 2 Department of Orthopedic Surgery, Bengbu Medical College, Bengbu, Anhui China; 3 Science and Technology Department, Lianyungang Second People’s Hospital Affiliated to Bengbu Medical College, The Second People’s Hospital of Lianyungang, 41 Hailian East Street, Lianyungang, 222003 Jiangsu China

**Keywords:** Percutaneous kyphoplasty, Osteoporosis, Fracture, Bone cement

## Abstract

**Background:**

Intravertebral cleft is common in osteoporotic vertebral compression fracture, and the bone sclerosis around the fissure brings difficulties to the surgical treatment. It is not known whether the balloon dilatation mode of percutaneous kyphoplasty affects the distribution of bone cement in the fracture vertebral body and further affects the surgical effect. The purpose of this study was to discuss the effect of balloon dilatation mode on percutaneous kyphoplasty in the treatment of osteoporotic vertebral fractures with intravertebral cleft.

**Methods:**

According to the inclusion criteria and exclusion criteria, a retrospective analysis of patients with osteoporotic vertebral fracture combined with intravertebral cleft treated by percutaneous kyphoplasty in our hospital was conducted. All patients were divided into two groups based on way of balloon dilation. The mode of balloon dilatation, imaging changes of vertebral body, VAS score, ODI score, bone cement distribution and postoperative complications were analyzed.

**Results:**

A total of 96 patients with osteoporotic vertebral fracture combined with intravertebral cleft were included in the study, including 51 patients treated with single balloon bilateral alternating dilatation technique and 45 patients treated with double balloon bilateral dilatation technique. The vertebral height, Cobb’s angle of kyphosis, VAS score and ODI score were significantly improved in both groups after operation (*P* < 0.05). The postoperative vertebral height and Cobb’s angle of kyphosis in the double balloon bilateral dilatation group were better than those in single balloon bilateral alternating dilatation group (*P* < 0.05). The distribution of bone cement in the single balloon bilateral alternating dilatation group was more inclined to insert filling, while the double balloon bilateral dilatation group was more inclined to fissure filling. The VAS score and ODI score at the final follow-up in the single balloon bilateral alternating dilatation group were lower than those in the double balloon bilateral dilatation group (*P* < 0.05).

**Conclusion:**

Double balloon bilateral dilatation technique can better restore the injured vertebral height in patients with osteoporotic vertebral fracture combined with intravertebral cleft. However, the distribution of injured vertebral cement in patients with single balloon bilateral alternating dilatation technique is more likely to be inserted and filled, and the long-term analgesia and lumbar function of patients are better.

## Background

Osteoporosis is a common metabolic bone disease [1, 2]. With the aging of the population, the incidence of osteoporosis increases year by year, and it is also the main cause of osteoporotic vertebral compression fracture. Osteoporotic vertebral compression fracture [3, 4] can lead to spinal deformity and low back pain, and the bone healing process is slow after fracture. The effect of conservative treatment of osteoporotic vertebral compression fracture is not good, and it is easy to cause more complications, such as pneumonia, lower limb deep venous thrombosis and so on. And another fracture may occur, resulting in disability or even death of the patient [5, 6]. Intravertebral cleft (IVC) is characterized by linear or cystic light transmission area on imaging, which is common in patients with osteoporotic vertebral compression fracture and has been widely concerned by scholars [7, 8]. Intravertebral cleft is generally considered to be an important factor leading to progressive vertebral collapse and kyphosis, intractable back pain and even spinal cord injury in patients with osteoporotic vertebral fracture [9].

Percutaneous kyphoplasty [10, 11] is a common minimally invasive operation for osteoporotic vertebral compression fractures. By placing balloons into the injured vertebrae and injecting bone cement after balloon dilatation and reduction of the fractured vertebrae, it can quickly stabilize the fractured vertebrae and relieve patients' pain. The mode of balloon dilatation is closely related to the distribution of bone cement in the injured vertebrae, which affects the clinical effect of percutaneous kyphoplasty [12]. By using the single balloon bilateral alternating expansion technique, the process of vertebral reduction can be observed slowly and dynamically, and the uneven force during unilateral excessive reduction and vertebral dilatation can be avoided, thus the risk of vertebral rupture during dilation can be reduced. Finally, serious complications caused by bone cement leakage can be effectively avoided [12]. Bilateral balloon dilatation at the same time can make the final plate uniform force, and better reduce the fractured vertebral body. For the osteoporotic vertebral fracture with intravertebral cleft, it is not known whether balloon dilatation will enlarge the intravertebral cleft, affect the distribution of bone cement and then affect the surgical effect. Therefore, this project takes the patients with osteoporotic vertebral fracture combined with intravertebral cleft as the research objects. Therefore, this project takes patients with osteoporotic vertebral fracture with vertebral fissure sign as the object, through a retrospective study of the effects of different balloon dilatation methods on spinal function, pain, morphological changes of fractured vertebral bodies, distribution of bone cement and postoperative complications, so as to provide a certain reference for clinical doctors to use balloon dilatation reasonably.

## Methods

### Inclusion criteria, exclusion criteria and grouping

Inclusion criteria: (1) Patients with fresh osteoporotic vertebral compression fracture were diagnosed by clinical manifestations and imaging data; (2) Combined with intravertebral cleft on X-ray and MRI; (3) The fractured vertebral body has a intact posterior wall of the vertebral body, without symptoms of spinal canal and nerve compression; (4) Patients with single vertebral lesion.

Exclusion criteria: (1) Vertebral posterior wall collapse defect accompanied by symptoms of dural sac or nerve tissue compression; (2) Vertebral infection or vertebral malignant tumor; (3) Patients with multiple vertebral fractures.

Grouping: group A: single balloon bilateral alternating dilatation technique; group B: double balloon bilateral dilatation technique.

### General information

According to the inclusion criteria and exclusion criteria, this study included 96 patients with osteoporotic vertebral fractures combined with intravertebral cleft treated by percutaneous kyphoplasty in our hospital from January 2018 to January 2021. According to the different ways of balloon dilatation, patients were divided into group A and group B. The age, sex and medical history of patients in each group were counted.

### Surgical methods

Group A: After disinfection and towel laying, local anesthesia was used. The bilateral transpedicular approach was selected and punctured into the injured vertebra through the pedicle under the guidance of X-ray machine, the working cannula was placed, and the balloon was inserted into the injured vertebra through the working cannula. Slowly expand the balloon, and then change to the opposite side of the working casing to expand at the same depth. When the kyphosis is corrected and the recovery of the vertebral body height is satisfied, stop the expansion and remove the balloon. Modulate the bone cement, and then inject the bone cement into the vertebral body. The injection process should be closely monitored, and stop after the dispersion is satisfied. After the bone cement hardened, the working channel was removed and covered with sterile excipients.

Group B: The operation process was the same as that of group A, but the balloon dilatation was performed by double balloon dilatation technique. The patient was implanted with a balloon after the establishment of a working passage through the bilateral pedicle. After the bilateral balloons were dilated to the fracture vertebral body at the same time, the bone cement was injected. After the bone cement hardened, the working channel was removed and the wound covered with aseptic excipients.

### Observation indicators

(1) Operation related index: Operation time and bone cement dosage of all patients were counted; (2) Imaging data: Measurement of anterior edge height of vertebral body and Cobb's angle of vertebral kyphosis before and after operation; (3) Clinical effect: Visual analogue score (VAS) and Oswestry Disability Index (ODI) were recorded before and after operation. (4) The distribution pattern of bone cement: According to whether the bone cement is fully embedded with the surrounding cancellous bone in the vertebral body, the distribution pattern of bone cement is divided into fissure-filling shape (Showing that the bone cement is confined in the fracture in the vertebral body. It is not fully intercalated with the surrounding cancellous bone) and intercalated-filling shape (Showing that bone cement can not only be fully filled in the fractures in the vertebral body. And well embedded in the surrounding cancellous bone). (5) Complications: The postoperative bone cement leakage, adjacent vertebral fracture and re-fracture were recorded.

### Statistical processing

SPSS23.0 software was used to analyze the data. The counting data were expressed by cases and percentages, and the comparison between groups was expressed by χ^2^ test, the measurement data was expressed by mean ± standard deviation, independent sample t test was used for comparison between groups, and paired t test was used for comparison within groups before and after operation. *P* < 0.05 as the difference was statistically significant.

## Results

### Comparison of basic information between the two groups

A total of 116 patients with osteoporotic vertebral fracture combined with intravertebral cleft were treated by percutaneous kyphoplasty in our hospital from January 2018 to January 2021. According to inclusion criteria, exclusion criteria and completeness of follow-up data, a total of 96 patients were included in the study, including 51 in group A and 45 in group B. The follow-up period of all patients was not less than 1 year.

The basic conditions of the two groups were compared in Table 1. There was no significant difference in age, sex, fracture section and bone mineral density between the two groups (*P* > 0.05). There was no significant difference in the amount of bone cement injection between the two groups (*P* > 0.05). However, the operation time and fluoroscopy times in group A were higher than those in group B (*P* < 0.05).

**Table 1:** Comparison of basic information

Classify		Group A	Group B	*P*-value
Age (years)		66.82 ± 9.87	67.31 ± 9.39	0.805
Follow-up time(months)		17.65 ± 3.79	18.13 ± 3.53	0.519
Gender	Male	17	12	0.478
	Female	34	33	
Bone mineral density		2.48 ± 0.59	2.43 ± 0.62	0.642
Preop. VAS		7.98 ± 0.88	8.02 ± 0.92	0.821
Preop. ODI		76.39 ± 7.97	77.33 ± 7.78	0.561
Operation time(min)		27.02 ± 4.75	22.47 ± 4.44	< 0.001
Fluoroscopy time		25.45 ± 4.74	21.82 ± 4.46	< 0.001
Cement volume(ml)		4.23 ± 1.17	4.54 ± 1.15	0.183
The fracture section		–	–	> 0.05
T9		1	1	–
T10		3	4	–
T11		11	8	–
T12		13	11	–
L1		11	10	–
L2		7	5	–
L3		2	4	–
L4		2	1	–
L5		1	1	–

*Pre-op* preoperative. Group A single balloon bilateral alternating dilatation technique. Group B double balloon bilateral dilatation technique

### Imaging evaluation

After operation and at the final follow-up, the height of anterior edge of vertebral body and Cobb’s angle of kyphosis were significantly improved in the two groups (*P* < 0.05). However, the anterior edge of vertebral body in group B was higher than that in group A and the Cobb’s angle of kyphosis in group B was lower than that in group A (*P* < 0.05) (Table 2).

**Table 2:** Comparison of imaging evaluation

	Classify	Pre-op	Post-op	Final follow-up	*P*-value
AHV	Group A	17.97 ± 2.51	21.85 ± 2.33	20.98 ± 2.40	< 0.001
	Group B	18.37 ± 2.36	23.21 ± 2.34	22.52 ± 2.48	< 0.001
	*P*-value	0.419	0.005	0.003	–
CVK	Group A	26.18 ± 4.40	16.89 ± 3.57	17.82 ± 3.60	< 0.001
	Group B	25.58 ± 4.62	14.75 ± 3.47	15.64 ± 3.35	< 0.001
	*P*-value	0.515	0.004	0.003	–

*Pre-op* preoperative, *Post-op* postoperative, *AHV* anterior margin height of vertebra, *CVK* Cobb’s angle of vertebral kyphosis, Group A single balloon bilateral alternating dilatation technique. Group B double balloon bilateral dilatation technique

### Comparison of clinical effects of surgery

The VAS score and ODI score of the two groups were significantly improved after operation and the final follow-up (*P* < 0.05). There was no significant difference in postoperative VAS score and ODI score between the two groups (*P* > 0.05). However, the VAS score and ODI score of group A were lower than those of group B at the final follow-up (*P* < 0.05) (Table 3).

**Table 3:** Comparison of VAS and ODI score

	Classify	Pre-op	Post-op	Final follow-up	*P*-value
VAS	Group A	7.98 ± 0.88	2.35 ± 0.74	1.78 ± 0.70	< 0.001
	Group B	8.02 ± 0.92	2.56 ± 0.69	2.96 ± 0.71	< 0.001
	*P*-value	0.821	0.172	< 0.001	–
ODI	Group A	76.39 ± 7.97	31.06 ± 5.11	22.98 ± 5.61	< 0.001
	Group B	77.33 ± 7.78	30.89 ± 5.27	30.13 ± 5.66	< 0.001
	*P*-value	0.561	0.873	< 0.001	–

*Pre-op* preoperative, *Post-op* postoperative. Group A single balloon bilateral alternating dilatation technique. Group B double balloon bilateral dilatation technique

### Comparison of distribution morphology of bone cement

In group A, the distribution of bone cement showed fissure-filling shape in 15 cases and intercalated-filling shape in 36 cases. In group B, the distribution of bone cement showed fissure-filling shape 25 cases and intercalated-filling shape in 20 cases. There was significant difference in the distribution of bone cement between the two groups (*P* < 0.05), and in Group A, the distribution of bone cement tended to be intercalated-filling shape, while in Group B, the distribution of bone cement tended to be fissure-filling shape.

### Comparison of postoperative complications between the two groups

In group A, there were 6 cases of bone cement leakage, including 3 case of intervertebral disc leakage and 3 case of paraspinal leakage. There were 8 cases of bone cement leakage in group B, including 3 cases of intervertebral disc leakage and 5 cases of paraspinal leakage. There was no significant difference in bone cement leakage between the two groups (*P* > 0.05). During the postoperative follow-up, there were 3 cases of re-fracture in group B and no re-fracture in group A.

## Discussion

This study compared the effects of single balloon bilateral alternating dilatation and double balloon bilateral dilatation on the clinical efficacy of percutaneous kyphoplasty in the treatment of osteoporotic vertebral fractures combined with intravertebral cleft. In terms of imaging, the vertebral height of patients treated with double balloon bilateral dilatation technique were higher than those treated with single balloon bilateral alternating dilatation technique. This may be related to the fact that the bilateral balloon dilatation technique can better expand the balloon uniformly so as to restore the height of the fractured vertebral body. However, in terms of the distribution of bone cement after operation, the distribution pattern of bone cement injected into the injured vertebrae by double balloon bilateral dilatation technique is more inclined to be confined in the cracks in the vertebral body and not fully intercalated with the surrounding cancellous bone. Balloon expansion compacted the loose bone trabeculae around the fissures in the vertebral body, making it form a tight bone shell around the balloon, which further prevented the cement from diffusing to the surrounding cancellous bone. The double balloon bilateral dilatation technique makes the bone around the balloon tighter, making it more difficult for the cement to disperse. So we think that balloon dilation affects the distribution of bone cement in the vertebra.

Although the lumbar function and pain of the two groups were significantly improved after operation, the final follow-up found that the lumbar function and pain of patients with double balloon bilateral dilatation were worse than those of patients with single balloon bilateral alternating dilatation. This is most likely due to the poor riveting of bone cement in injured vertebrae and surrounding bone in patients with bilateral dilatation of double balloons. As time goes by, there is a fretting between bone cement and surrounding bone, resulting in poor lumbar function and pain in patients.

On the other hand, we believe that the reason for the different effects of pain and lumbar function in the two groups may be related to the distribution of bone cement after surgery. A large number of clinical studies have shown that the clinical effect of percutaneous kyphoplasty is closely related to the distribution of bone cement [13, 14]. When percutaneous kyphoplasty is used to treat osteoporotic vertebral fracture without intravertebral cleft, the injected bone cement filled in the space of injured vertebral cancellous bone with spongy structure has better biomechanics [15]. For patients with osteoporotic vertebral fracture combined with intravertebral cleft, when bone cement is injected during the operation, the internal of the fracture in the vertebral body is in a state of negative pressure like a “reservoir”, and the injected bone cement will be filled in the fissure area in the shape of a solid mass. At the same time, due to the blocking of the fibrous membrane and hardened bone around the fissure area, the massive bone cement in the intravertebral cleft is limited to spread to the surrounding cancellous bone area. The stiffness and strength of the massive cement in the intravertebral cleft of the vertebral body are significantly higher than that of the surrounding cancellous bone. When bone cement can’t or rarely carry out insertion with the surrounding cancellous bone, it will cause compression to the peripheral fragile cancellous bone, resulting in a decrease in the stability between cement and cancellous bone, thus weakening its biomechanical properties and affecting the long-term clinical effect [16]. This may be the reason why lumbar function and pain were worse in patients with double balloon bilateral dilatation at the last follow-up than in patients with single balloon bilateral alternating dilatation.

The double balloon bilateral dilatation technique can reduce the operation time, the number of intraoperative X-ray fluoroscopy and better restore the height of the vertebral body compared with single balloon bilateral alternating dilatation technique. However, in terms of clinical efficacy and cement distribution, single balloon bilateral alternating dilatation technique can make the bone cement in the injured vertebrae better embedded into the surrounding cancellous bone, with a better long-term effect. However, this study has some limitations, because it is a retrospective study, it can’t fully demonstrate the effects of factors such as the size of vertebral fissures, the contents of fissures and the viscosity of bone cement on the effect of operation. Due to the low incidence of osteoporotic vertebral compression fracture patients combined with intravertebral cleft and in order to eliminate the interference of other factors as far as possible, we adopted more stringent inclusion criteria. The sample size is relatively small, so multicenter, large sample size prospective studies are needed to further verify our conclusions.

## Data Availability

The datasets generated or analysed during the current study are not publicly available due there were other research plan in future but are available from the corresponding author on reasonable request.
